# Bioinspired Composite, pH-Responsive Sodium Deoxycholate Hydrogel and Generation 4.5 Poly(amidoamine) Dendrimer Improves Cancer Treatment Efficacy via Doxorubicin and Resveratrol Co-Delivery

**DOI:** 10.3390/pharmaceutics12111069

**Published:** 2020-11-09

**Authors:** Tefera Worku Mekonnen, Abegaz Tizazu Andrgie, Haile Fentahun Darge, Yihenew Simegniew Birhan, Endiries Yibru Hanurry, Hsiao-Ying Chou, Juin-Yih Lai, Hsieh-Chih Tsai, Jen Ming Yang, Yen-Hsiang Chang

**Affiliations:** 1 Graduate Institute of Applied Science and Technology, National Taiwan University of Science and Technology, Taipei 106, Taiwan; tefe16@gmail.com (T.W.M.); habegaz21@gmail.com (A.T.A.); fentahunhailebdu@gmail.com (H.F.D.); yihenews@gmail.com (Y.S.B.); idrisbrhm7@gmail.com (E.Y.H.); wherelove8@gmail.com (H.-Y.C.); jylai@mail.ntust.edu.tw (J.-Y.L.); 2 Advanced Membrane Materials Center, National Taiwan University of Science and Technology, Taipei 106, Taiwan; 3 R & D Center for Membrane Technology, Chung Yuan Christian University, Chungli, Taoyuan 320, Taiwan; 4 Department of General Dentistry, Chang Gung Memorial Hospital, Taoyuan 333, Taiwan; Cyh4d25@adm.cgmh.org.tw; 5 Department of Chemical and Materials Engineering, Chang Gung University, Taoyuan 333, Taiwan

**Keywords:** codelivery, doxorubicin, G4.5 poly(amidoamine), resveratrol, sodium deoxycholate hydrogel

## Abstract

Maximizing the antitumor efficacy of doxorubicin (DOX) with a new drug delivery strategy is always desired in the field of biomedical science. Because the clinical applications of DOX in the treatment of cancer is limited by the side effects related to the dose. Herein, we report the co-loading of DOX and resveratrol (RESV) using an injectable in situ formed sodium deoxycholate hydrogel (Na-DOC-hyd) at the pH of the tumor extracellular microenvironment. The sequential, controlled, and sustained release of RESV and DOX for synergistic antitumor effects was confirmed by entrapping G4.5-DOX in the RESV-loaded Na-DOC hydrogel (Na-DOC-hyd-RESV). The synergistic antitumor activity of Na-DOC-hyd-RESV+G4.5-DOX was assessed on HeLa cell xenograft tumor in BALB/c nude mice. In the MTT biocompatibility assay, both the G4.5 PAMAM dendrimer and Na-DOC-hyd exhibited negligible cytotoxicity up to the highest dose of 2.0 mg mL^−1^ in HeLa, MDA-MB-231, and HaCaT cells. The release profiles of DOX and RESV from the Na-DOC-hyd-RESV+G4.5-DOX confirmed the relatively rapid release of RESV (70.43 ± 1.39%), followed by that of DOX (54.58 ± 0.62%) at pH 6.5 in the 7 days of drug release studies. A single intratumoral injection of Na-DOC-hyd-RESV+G4.5-DOX maximally suppressed tumor growth during the 28 days of the treatment period. Na-DOC-hyd-RESV+G4.5-DOX did not cause any histological damage in the major visceral organs. Therefore, this Na-DOC-hydrogel for dual drugs (DOX and RESV) delivery at the pH of the tumor extracellular microenvironment is a promising, safe, and effective combination for antitumor chemotherapy.

## 1. Introduction

Cancer is the most complicated disease affecting mankind. As cancer has now become a global health issue, cancer-related studies of the past few decades have mainly focused on improving the efficacy [1] and minimizing the limitations of the currently applicable therapeutic drugs such as cisplatin and doxorubicin [2] Combination chemotherapy is the most applicable approach to combat cancer by combining two or more therapeutic drugs [3,4,5,6] Compared to the single drug therapeutic approach, the co-delivery of anti-cancer drugs enhances cancer cell killing efficacy, the use of two or more drugs together or in a sequential manner may enable targeting of several key pathways involved in cancer cell sustenance and proliferation in a synergistic or additive manner [3].

DOX is one of the most potent anticancer drugs used for treating different forms of cancer, however its clinical application is limited by the dose-dependent side effects [2,7]. The most frequently reported undesirable effects of DOX include congestive heart failure [8], reversible nephrotoxicity [9], and irreversible degenerative cardiomyopathy [2,10]. Thus, several studies have investigated methods of mitigating DOX cardiomyopathy and other adverse effects by combining it with other chemotherapeutic drugs and phytochemicals.

Phytochemicals, compounds produced by plants, such as curcumin [11], piperine [12] and resveratrol (RESV) [13] are frequently used in combination with DOX [14]. Since the last few years, combinations of DOX with phytochemicals have attracted the attention of oncologists. Because phytochemicals have reported to enhance chemo-sensitivity or therapeutic efficacy and limit the undesired effects of drugs DOX [15].

Resveratrol is naturally occurring phytochemical that has attracted the attention of the scientific community due to its diverse biological importance such as antioxidant [15], anti-inflammatory [16], anti-cancer [17], and the ability to reverse the multidrug resistance in cancer cells [18]. In combination with clinically known drugs RESV can enhance sensitization of cancer cells to the standard drugs [13,19]. However, RESV delivery to the target site is challenging due to its poor solubility, bioavailability, and rapid metabolism with short half-life. Hence, RESV has been conjugated with or encapsulated in drug carrier system for enhancing its pharmacokinetic profile [17]. Dendrimers are nanometer size synthetic polymer with a highly branched structures and globular shape that can improve the stability and bioavailability of RESV by encapsulating their interior void space [20]. Among dendrimers, the poly(amidoamine) (PAMAM) polymer has received attention for its efficacy as potential drug delivery system [21,22].

However, owing to the safety issue related to the nano meter size and surface charge of the PAMAM dendrimer, prolonged application may result in adverse effects or toxicity to visceral organs such as the kidney, spleen, liver, lung, and heart. particularly, PAMAM dendrimers with cationic and amine functional surface are more toxic than their anionic counterparts [23]. Each dendrimer exhibits a characteristic drug release profile. For instance, dendrimers with -COOH surface group showed more rapid drug release than those with NH_2_ surface group [23]. Therefore, formation of drug-loaded dendrimers entrapped in another drug-loaded hydrogel can achieve a therapeutically desired effect, as the hydrogel can reduce the rapid release of the drugs from the dendrimer, provide high drug loading capacity, and ensure sustainable and sequential release of the drugs. Sustained and controlled release of the co-loaded drugs can be achieved due to relatively slow degradation and high drug loading capacity of the hydrogel [24].

Low molecular weight natural substances have attracted considerable attraction as good alternatives to polymeric biomaterials for developing hydrogels for drug carrier systems. The major advantages of using low molecular weight biosurfactants for the formation of hydrogel over synthetic polymers is the former’s low melt viscosity high biodegradability [25], high biocompatibility, and the absence of toxic impurities due to simple preparation protocol [26]. Sodium deoxycholate (Na-DOC) bile salt is a naturally occurring low molecular weight substance that can form hydrogel [27,28,29]. The Na-DOC molecules can form macro gel in the presence of inorganic salts (NaBr and NaCl) [29,30,31] and amino acids [30,32].

We believe this study is the first to develop a bioinspired Na-DOC hydrogel for sequential and sustainable dual anticancer drugs delivery. In this study we aimed to develop in situ sol-to-gel phase transition of sodium deoxycholate hydrogel (Na-DOC-hyd) for dual drugs delivery at the pH of the tumor extracellular microenvironment. During the preparation mannitol (Mnt) and NaCl were used as gel modifier and cross linker, respectively. We have optimized the content of Mnt/NaCl for stable gel formation of the Na-DOC for intratumoral drugs delivery. The sequential and sustained release of dual drugs for synergistic antitumor activity was attained by entrapping drug loaded PAMAM dendrimer NPs in hydrogel. So that the drug loaded in the polymer released slowly and delayed few minutes or hours after release of the drug from the hydrogel. Whether the Na-DOC gel can be used as a drug carrier system warrants further investigations. We believe that this novel in-situ sol-to-gel phase transition of Na-DOC hydrogel can be used clinically for sustainable and sequential anticancer dual drugs release at tumor extracellular microenvironment (pH 6.5).

## 2. Materials and Methods

### 2.1. Materials

Sodium deoxycholate bile salt (Na-DOC ≥ 98%), NaCl, Paraformaldehyde and Dimethyl sulfoxide (DMSO), Absolute Ethanol (98%) purchased from Sigma, Mannitol(Mnt) (St. Louis, MO, USA) was from Merck (Darmstadt, Germany), Carboxyl-terminated G4.5-poly(amidoamine) was purchased from Dendritech Inc. (Midland, MI, USA). Dulbecco’s modified Eagle medium (DMEM), penicillin, sodium pyruvate, trypsin, and fetal bovine serum (FBS) were purchased from Gibco (Carlsbad, CA, USA). HaCaT (keratinocyte cell line from adult human skin) and MDA-MB 231 and HeLa cells (human cervical carcinoma cell) were obtained from the BioResource Collection and Research Center (Hsinchu, Taiwan), Sterilized phosphate-buffered saline (PBS), 3-(4,5-dimethylthiazol-2-yl)2,5-diphenyl-tetrazolium bromide (MTT) were purchased from Gibco (Carlsbad, CA, USA). Deionized water used in this study was purified with a Milli-Q Plus 185 system (Millipore, Burlington, MA, USA) and had resistivity >18.2 MΩ-cm. Doxorubicin hydrochloride (DOX. HCl) and Resveratrol (RESV) were purchased from Cayman Chemical Co., Ltd. (Ann Arbor, MI, USA) and Sigma Aldrich (St. Louis, MO, USA), respectively. All chemicals and organic solvents were of analytical grade.

### 2.2. Preparation of In Situ Sodium Deoxycholate Gelling Systems

The *in-situ* sodium deoxycholate hydrogel (Na-DOC-hyd) was prepared in the pH of the tumor extracellular microenvironment for anti-cancer drug delivery. The Mnt and NaCl were used as gel modifying and cross-linking agents, respectively. Various but equal concentration (in mmole L^−1^) ratios of Mnt and NaCl have been used to optimize the Na-DOC sol-to-gel phase transition in 0.1 M PBS at pH ranging from 6.5 to 6.7. Briefly, a mixture of Na-DOC (60 mmole L^−1^) with various Mnt/NaCl ratios were mildly sonicated at 37 °C for 10 min. The mixtures were transferred into vials for overnight incubation at room temperature (22 ± 3 °C). The phase transition and stability of the gels were assessed via test tube inversion method. For the test tube inversion study, 3 mL at sol state of the prepared solutions was injected into a 20 mL vial with an inner diameter of 47.6 mm and placed overnight at 22 ± 3 °C [31].

### 2.3. Characterization Na-DOC-Hyd

Some selected samples were further characterized based on the information obtained from the test tube inversion study. The techniques used for characterization of the selected samples were rheology, attenuated total reflectance (ATR) spectroscopy (JASCO FTIR-6700, Neuchatel, Switzerland), X-ray diffraction (XRD), (Kyoto, Japan), and field-emission scanning electron microscopy (FESEM) (JSM 6500F, JEOL, Peabody, MA, USA).

#### 2.3.1. Rheological Study of Na-DOC-Hyd

Rheological studies of the gels were conducted to investigate the viscoelastic properties and oscillatory measurements (elastic modulus and viscous modulus) using Modular Compact Rheometer (MCR102, Graz, Austria) with a cone-plate system at 37 °C. Oscillatory measurements were conducted in a frequency range of 0.01–10 Hz. The complex modulus (G*) of each of the selected samples were determined before oscillatory frequency sweep test. The samples selected for rheological studies were consist of 35, 45 and 50 mmole L^−1^ concentration of Mnt/NaCl.

#### 2.3.2. Studies on Na-DOC-Hyd Morphology

The morphology and the pores nature of hydrogels samples were examined using field-emission scanning electron microscopy (FESEM) (JSM 6500F, JEOL). Briefly, three hydrogel samples prepared with 35, 45 and 50 mmole L^−1^ Mnt/NaCl were immersed in liquid nitrogen for 20 min and then lyophilized with freeze dryer for 24 h. Then, 1 mg of the dried samples were placed on silicon wafer substrates and coated with platinum for 10 min for capturing images.

#### 2.3.3. Na-DOC-Hyd X-ray Diffraction Studies

The same samples used in the FESEM study were also examined to determine their diffraction pattern at 22 ± 3 °C in the range of 10° to 80° at 2θ scan mode (2.5° min^−1^) using a diffractometer with a Cu-Kα radiation source. Sample prepared with 50 mmole L^−1^ Mnt and 60 mmole L^−1^ without NaCl were also tested to compare crystallinity and assess the effect of NaCl on gel microstructure.

#### 2.3.4. Na-DOC-Hyd Fourier Transform Infrared (FTIR) Studies

FTIR was used to further study the effect of Mnt on hydrogen bond formation in the gelation process of Na-DOC molecules. The FTIR spectra of the dried samples were recorded by attenuated total reflectance (ATR) spectroscopy (JASCO FTIR-6700; Jasco, Easton, MD, USA).

#### 2.3.5. Swelling Ratio of Na-DOC-Hyd

The swelling ratio of gel samples prepared with 35, 45, and 50 mmole L^−1^ Mnt/NaCl were examined. The swelling ratio of the hydrogels were determined using the gravimetric approach [33]. Known weights of the gel samples were immersed in 0.1 M PBS at pH 6.5 and 37 °C for few hours until the equilibrium point was reached. Then, excess PBS not immobilized on the surface was gently wiped off using soft filter paper and the samples were reweighed. The measurements were made in triplicates. The swelling ratio was calculated using the following formula

(1) $$\mathrm{Swelling}\mathrm{ratio}\left(\%\right)=\frac{\left(\mathrm{Ws}-\mathrm{Wd}\right)}{\mathrm{Wd}}\times 100\;$$

where ‘Ws’ is the mass of the swollen hydrogel and ‘Wd’ is the mass of the dry powder sample.

#### 2.3.6. Degradation of Na-DOC-Hyd

The solid tumor and its adjacent tissues are acidic. Hence, the degradation behavior of the hydrogel used in swelling studies was also evaluated in acidic microenvironments (pH 5.0 and 6.5) at 37 °C. A known weight of hydrogel (Wo) was initially added into each of a 7 mL vials. Then, the vials were incubated for 3, 6, 12, 24, 48, 72, 96, 120, 144 and 168 h using a 0.1 M PBS. After the hydrogels underwent degradation at the appropriate time, the PBS was gently removed and the residual hydrogels inside the vials (Wt) were weighed. At predetermined time points, the ratio of weight loss to the initial dry weight of the samples were estimated. The rate of weight loss of the hydrogel was estimated using the following equation [3,34].

(2) $$\mathrm{Weight}\mathrm{loss}\left(\%\right)=\frac{\mathrm{Wo}-\mathrm{Wt}}{\mathrm{Wo}}\times 100$$

Measurements (in triplicate) of both the degradation and swelling results at each of the predetermined time were performed and the results were expressed as mean ± standard deviation (SD).

### 2.4. Drug Loading and Releasing Studies

#### 2.4.1. Doxorubicin Loading and Releasing of G4.5 PAMAM Dendrimer

Doxorubicin encapsulation in G4.5 PAMAM dendrimer (G4.5-DOX) was performed based on previously reported method with some modifications [35,36,37,38]. Briefly, 10 mg G4.5 PAMAM was dissolved in 2 mL deionized water. DOX (1 mg) powder was dissolved in 0.60 mL dimethyl sulfoxide (DMSO). The acidic DOX solution was neutralized with 5 μL triethylamine. The DOX solution was added dropwise to the G4.5 PAMAM dendrimer solution and the mixture was vigorously stirred for 12 h. Then, the mixed solution was dialyzed in deionized water for 36 h using a membrane with 1 kDa MW cut-offs to remove unencapsulated DOX. The drug-encapsulation efficiency (DEE) (Equation (3)) and drug-loading capacity (DLC) (Equation (4)) were calculated by analyzing the UV–vis spectra at 490 nm [36]. The calibration curve for free DOX was plotted at 484 nm.

(3) $$\mathrm{DEE}=\frac{\mathrm{Amount}\mathrm{of}\mathrm{Drugs}\mathrm{in}\mathrm{Nanoparticles}}{\mathrm{Initial}\mathrm{amount}\mathrm{of}\mathrm{feeding}\mathrm{Drugs}}\times 100$$

(4) $$\mathrm{DLC}=\frac{\mathrm{amount}\mathrm{of}\mathrm{feeding}\mathrm{drugs}-\mathrm{amount}\mathrm{of}\mathrm{non}-\mathrm{loaded}\mathrm{drugs}}{\mathrm{Weight}\mathrm{of}\mathrm{the}\mathrm{drug}-\mathrm{loaded}\mathrm{NPs}}\times 100$$

#### 2.4.2. Co-Encapsulation of RESV and G4.5-DOX in Na-DOC-Hyd

The lyophilized Na-DOC-hyd (50 mg) dissolved in 1.0 mL 0.1 M PBS (pH = 6.5) was mixed with 10 mg RESV dissolved in warm water. The RESV-loaded hydrogel (Na-DOC-hyd-RESV) was obtained after 5 min of sonication. Then, G4.5-DOX was entrapped, followed by mixing with Na-DOC-hyd-RESV in the sol state [39]. Briefly, 10 mg G4.5-DOX and 1.67 mL Na-DOC-hyd-RESV were mixed and sonicated for 5 min with 40% power and 2 s gap time (JY92-IIN, Ultrasonic Homogenizer Ningbo, China).

### 2.5. Drug Release Studies of G4.5-DOX and Na-DOC-Hyd-RESV+G4.5-DOX

The release pattern of DOX from G4.5-DOX and that of RESV and DOX from Na-DOC-hyd-RESV+G4.5-DOX were tested in media of different pH (5.0, 6.5 and 7.4). DOX release studies from G4.5-DOX were performed based on a previously reported method [35,36,37,38,40]. Briefly, 1.0 mL G4.5-DOX solution was placed in a dialysis tube (membrane with 1 kDa MW cut-offs) and immersed in a separate vial containing 10 mL PBS at various pH (5.0, 6.5 and 7.4).

The drug release studies of Na-DOC-hyd-RESV+G4.5-DOX were performed using dialysis method. Briefly, 1.5 mL Na-DOC-hyd-RESV+G4.5-DOX bound to the 1 kDa MW cut-offs membrane was inserted in to a 20 mL vial. Then, 4 mL PBS at 7.4 and adjusted PBS at pH of 5.0 and 6.5 were added separately as drugs releasing media and incubated at 37 °C with mild shaking (60 RPM) in the shaking machine [41].

At predetermined time-interval 3 mL of aliquots from the releasing media were collected gently and equal amount of fresh PBS with the corresponding pH was replenished. The amount of released drug was estimated from the collected release media using UV–Vis spectrophotometer at 499 nm and 320 nm wavelength for DOX and RESV, respectively. The cumulative percentage of drug-release was calculated based on the calibration curve prepared for free drugs (Figure S1a–d).

### 2.6. In Vitro Cytotoxicity Test (MTT Assay)

MTT assay for determining the biocompatibility of Na-DOC-hyd and G4.5-PAMAM dendrimers were conducted on multiple cell lines (MDA-MB-231, HaCaT, and HeLa). The cells were incubated for 24 h with G4.5 dendrimer and Na-DOC-hyd. Briefly, 5 × 10^3^ cells were seeded per well on a 96-well plate in a complete DMEM containing 1% (*w*/*v*) penicillin, 1% (*w*/*v*) sodium pyruvate, 1% (*w*/*v*) glutamine, and 10% (*w*/*v*) FBS. The cells were cultured at 37 °C and 5% of CO_2_ adjusted incubator. The cells were incubated for 24 h with empty G4.5 PAMAM dendrimer and Na-DOC-hyd. Then, the old media was removed and the cells were washed thrice, followed by the addition of 20 μL MTT assay solution (5 mg mL^−1^) mixed with DMEM and incubated for additional 4 h at 37 °C and a humidified atmosphere of 5% CO2. Subsequently 100 µL DMSO was added and incubate for 30 min to dissolve the formed formazan complex. The percentage of viable cells were estimated using absorbance values at 570 nm in an enzyme linked immunosorbent assay (ELISA) reader (Multiskan FC, Microplate Photometer; Thermo Fisher Scientific, Waltham, MA, USA). The cytotoxicity was determined as the relative percentage of viable cells in the treatment group compared to that in the control group using the formula mentioned below.

(5) $$\mathrm{Cell}\mathrm{viability}\left(\%\right)=\frac{\mathrm{Absorbance}\mathrm{of}\mathrm{treated}\mathrm{cells}}{\mathrm{Absorbance}\mathrm{of}\mathrm{control}\mathrm{cells}}\times 100$$

### 2.7. Cellular Uptake Studies

The intracellular drug release and cellular uptake from G4.5-DOX and Na-DOC-hyd-RESV were qualitatively assessed by comparing the intensity of the red (DOX) and green (RESV) fluorescence emitted by the drugs incorporated by HeLa cells with that of the free drugs (control) (using iRiS™ Digital Imaging System from Logos Biosystems, Annandale, VA, USA). The HeLa cells were seeded in DMEM supplemented with 1% (*w*/*v*) penicillin, 10% (*w*/*v*) FBS and incubated at 37 °C in a humidified atmosphere of 5% CO2. The HeLa cells were grown to a density of 1 × 10^5^ mL^−1^ on a coverslip in a 33 mm confocal dish for 24 h. Then again, the cells were co-incubated with free drugs (DOX or RESV, or RESV+DOX) and G4.5-DOX or Na-DOC-RESV at equivalent DOX and RESV concentration of 2.5 μg mL^−1^ and 7.5 μg mL^−1^, respectively. The cells were incubated for about 2, 4 and 8 h with G4.5-DOX NPs and 4, 8 and 12 h with Na-DOC-hyd-RESV. Subsequently, the dishes were emptied, washed thrice with 0.1 M PBS and stained with 500 µL 4′,6-diamidino-2-phenylindole (DAPI) (0.3 μM) for 15 min, followed by fixing with 4% (*w*/*v*) paraformaldehyde for another 20 min.

### 2.8. Animal Experiment

The synergistic antitumor efficacy of the co-loaded DOX and RESV using the G4.5 PAMAM dendrimer entrapped in RESV-loaded Na-DOC-hydrogel was evaluated on cancer cells xenografted tumor in BALB/c nude mice. The six-week-old female BALB/c mice (weighing 19 ± 0.76 g) were purchased from BioLASCO Taiwan Co., Ltd., Taipei, Taiwan). The mice were handled, and all the animal experiments were performed according to the guidelines developed by the institutional animal handling and research committee at National Taiwan University. The mice were maintained in standard pathogens-free cages under natural light/dark conditions at room temperature in an atmosphere of 55 to 60% relative humidity; they were supplied with bedding and provided *ad libitum* access to a regular chow and tap water. After 7 days of acclimatization with the environment, the HeLa cells xenografted tumor was developed via subcutaneous injection of a 0.1 mL of HeLa cells (2 × 10^6^) suspension in DMEM into the right flank of each mouse. To prevent multiple tumor formation, cells were evenly inoculated into the pouch formed by the fingers. The tumor volumes (TV) and body weights were measured every other day. The tumor volumes were calculated using the following equation:

(6) $$\mathrm{Tv}({\mathrm{mm}}^{3})=\frac{l\times w^{2}}{2}$$

where ‘*l’* and ‘*w’* are the larger diameter (length) and the shorter diameter (width) of the tumor, respectively.

After tumors size reached about 100 to 150 mm^3^, the mice were randomly distributed into six groups (four mice per group) and different treatments formulations were administered: group 1: physiological saline solution; group 2: Na-DOC-hyd+G4.5 PAMAM; group 3: Na-DOC-hyd-RESV; group 4: Na-DOC-hyd+G4.5-DOX; group 5: Free DOX/RESV in saline; group 6: Na-DOC-hyd-RESV+G4.5-DOX (all drug containing formulations prepared at equivalent DOX and RESV concentration of 10 mg kg^−1^ and 30 mg kg^−1^, respectively).

The tumor volume and body weights were measured every other day of the treatment period (28 days). The tumor volume inhibition rate (TVIR) used to evaluate the antitumor efficacy of the different treatment formulations. TVIR was calculated using the following formula:

(7) $$\mathrm{TVIR}\left(\%\right)=\left[\frac{\mathrm{vt}-\mathrm{vc}}{\mathrm{vc}}\right]\times 100$$

where ‘vc’ and ‘vt’ represent the mean tumor volume of the control and treatment group, respectively after 28 days of treatment period.

### 2.9. Histopathological Studies

After 28 days of the treatment, the mice were euthanized by cervical dislocation and the major internal organs (lung, heart, liver, spleen, and kidney) and tumor tissues were collected. Then histopathological and immunohistochemical staining were performed based on the protocol reported previously [3]. Briefly, the organs and tumors tissues were fixed in 4% paraformaldehyde and embedded in paraffin-wax then they were serially deparaffinized and rehydrate, followed by staining of the 5 µm thick sectioned samples with hematoxylin (H) and eosin (E). Next, dehydration with absolute alcohol and xylazine and mounting with gum arabic were performed to assess the histological change. In addition, tumors were immunohistochemically stained with CD31 to determine the size of blood vessels (diameter) and micro-vessel density (MVD). Digital images of the micro-vessels were assessed from the tumor samples and the densities were blindly evaluated using MDSAS software (Motic Digital Slide Assistant System Lite 1.0, New York, NY, USA).

### 2.10. Statistical Analysis

All the numerical data were measured at least in triplicate, and the data were explained as the mean ± standard deviation. The experimental groups were compared using analysis of variance (ANOVA) using the Origin software. Differences were significant at the *p* < 0.05 (*). Probabilities of *p* < 0.01 (**) and *p* < 0.001 (***) were considered as highly significant.

## 3. Results and Discussions

### 3.1. Preparation and Characterizations of Na-DOC-Hyd

**Figure 1:**
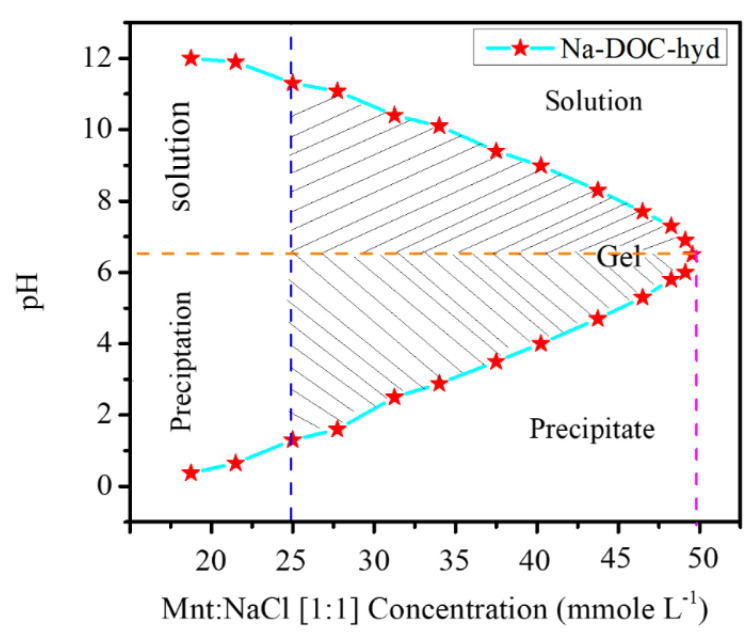
Optimizing the concentration of Mnt (mannitol) and NaCl for in situ gelation of sodium deoxycholate (Na-DOC) in the tumor extracellular microenvironment (pH 6.5) (Mnt: (1:1 mmole L^−1^ concentration ratios of Mnt: NaCl were used).

We confirmed that the formation of the in situ injectable Na-DOC hydrogel as a cancer drug carrier at the tumor extracellular microenvironment. The concentrations of Mnt and NaCl used as gel modifying and cross-linking agents during the insitu gel formation of the Na-DOC were identified. During optimization, 1:1 mmole L^−1^ concentration ratios of Mnt/NaCl were used. The phase transition of samples in each 5 mmole L^−1^ Mnt/NaCl concentration interval was assessed using the test tube inversion method. As shown in Figure 1, gel features of the prepared samples were observed in those solutions containing 20–50 of Mnt/NaCl mmole L^−1^. However, gels containing 20, 25, and 30 mmole L^−1^ Mnt/NaCl were not selected for further studies, as these gels were relatively unstable.

**Figure 2:**
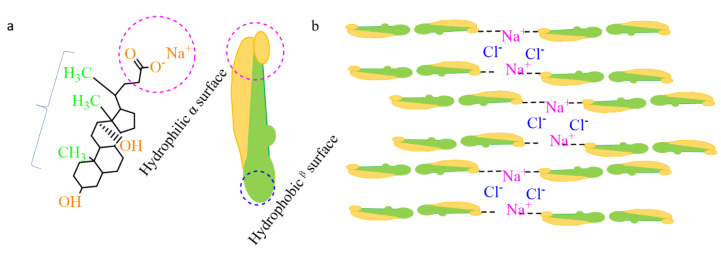
Schematicrepresentation of (**a**) the srructure of Sodium deoxycholate(Na-DOC) and (**b**) Sodium deoxycholate hydrogel formation induced by NaCl.

According to previous studies, the formation of a complex network via the self-assembly of the Na-DOC molecules were due to noncovalent interactions, such as metal coordination, hydrogen bonding, electrostatic interactions, and hydrophobic effect (Figure 2a,b) [31,42,43]. Na-DOC is a low molecular weight substance that forms hydrogels in aqueous solution due to entanglement micellization. However, the hydrogels we developed are thixotropic in nature with less mechanical properties. The contribution of gel modifiers such as mannitol and Tris buffer to hydrogen bonding during gelation of Na-DOC has been reported [44,45]. Halide salts (NaCl, NaBr) as cross-linkers have been shown to improve the poor mechanical properties of the thixotropic Na-DOC gels [45,46], as the ions from the salts compressed the thick electric double layer and reduced the electrostatic repulsion of adjacent Na-DOC molecules at their polar heads [42]. As the mechanism illustrated in Figure 2b the weak coordination bonds formed between the Na^+^ ions and the carboxylate(-COO-) polar head of the Na-DOC molecules, and the electrostatic interaction due to Cl^−^ and α-methylene moiety attached to the carboxylate group of the bile salt are also involved in the gelation of Na-DOC molecules [42,46].

In the Na-DOC pH responsive hydrogel preparation study, we also confirmed the effect of Mnt/NaCl on enhancement of the mechanical properties of the in-situ Na-DOC gel at the pH of the cancer extracellular microenvironment. The test tube inversion study revealed that the mechanical properties such as consistency and stability of gels improved with increasing Mnt/NaCl concentration. Per the observation of the results obtained during the optimization and test tube inversion method, gel samples prepared with 35, 45, and 50 mmole L^−1^ Mnt/NaCl were selected for further characterization (Figure 1 and Figure S1). The gel with 50 mmole L^−1^ Mnt/NaCl was the thickest and most stable.

#### 3.1.1. Rheological Study of the Na-DOC-Hydrogel

We examined the viscoelastic properties such as oscillatory measurements and frequency sweep test of gels using rheological parameters. But before the oscillatory measurements, we confirmed that the selected shear stress was in the linear viscoelastic region. As shown in Figure S3, the linear viscoelastic regions of the three different gel samples were different. All the samples tested were within the linear viscoelastic region, where the complex modulus (G* or G star, in Pa is the entire viscoelastic behavior of a sample) (G*) was independent of the applied stress [47]. The widest linear viscoelastic region of the gel containing 50 mmole L^−1^ Mnt/NaCl is due to the overlap of the Na-DOC molecular chains, which enables them to withstand high shear stress and delays the destruction of the gel microstructure [31].

**Figure 3:**
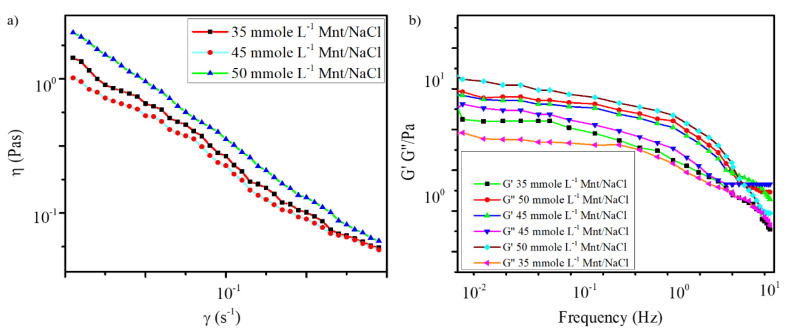
(**a**) Variation of shear viscosity as a function of shear rate (**b**) Variation of G′ and G″ as a function of frequency for gels with 60 mmole L^−1^ sodium deoxycholate(Na DOC) and 35, 45 and 50 mmol L^−1^ Mnt/NaCl (Mnt/NaCl concentration ratio was 1:1 mmole L^−1^).

The oscillatory measurements were performed in the frequency range of 10^−1^–10^1^ Hz to determine the viscoelastic properties of the samples with three different concentrations of Mnt/NaCl. As shown in Figure 3a, the apparent viscosity (η) of the gels also improved with increase in Mnt/NaCl concentration. This showed that the use of 50 mmole L^−1^ Mnt/NaCl improved the stiffness and viscoelasticity of the gels [31,48].

Per the result of the frequency sweep test shown in Figure 3b, the “elastic modulus” G′ of all the tested samples was higher than the “viscosity modulus” G″ at a lower frequency, indicating that the elastic component was dominant. Both G′ and G″ decreased with increasing frequency, and above a critical frequency, G″ exceeded G′, indicating that the system became more viscous than elastic [45]. Furthermore, both the elastic and viscous modulus curves of the 50 mmole L^−1^ Mnt/NaCl hydrogel sample were located above those of all the other samples, indicating that the highest Mnt/NaCl content (50 mmole L^−1^) had improved the mechanical strength of the gel [45]. In addition, the tested samples exhibited shear-thinning trend, as shown in the shear flow curves of Figure 3b. The results of the rheological study confirmed the observations of the test tube inversion experiment.

#### 3.1.2. Microstructural Study of Na-DOC-Hyd Using XRD

**Figure 4:**
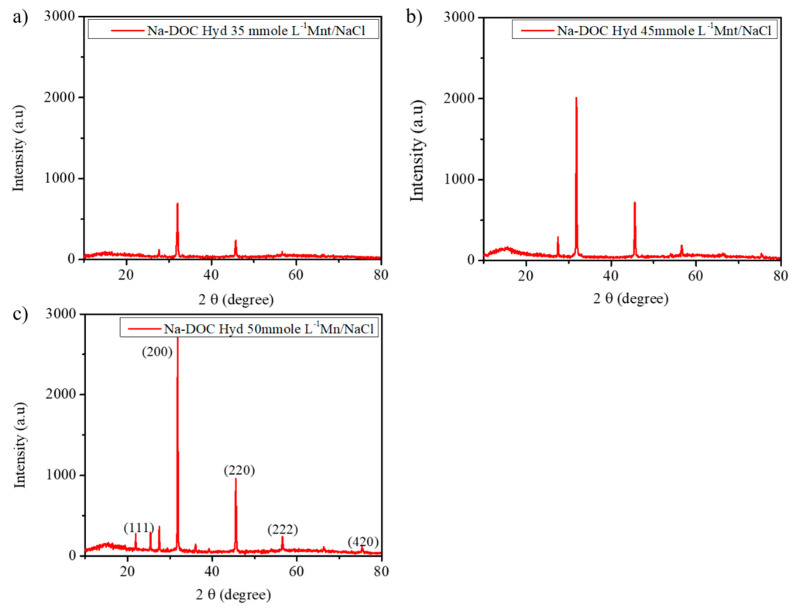
XRD patterns of the gels with 60 mmole L^−1^ sodium deoxycholate (Na-DOC) and 35, 45 and 50 mmole L^−1^ Mnt/NaCl (1:1 mmole L^−1^ concentration ratio of Mnt/NaCl used).

To obtain further information regarding the structural attributes of the hydrogels, we used XRD spectra to assess the self-assembly of the Na-DOC molecules. A previous study indicated that several factors, including the concentrations of the gel modifying and gel cross-linking agents and temperature, determine the nature of the microstructure and the arrangement of Na-DOC molecules during gel formation [31] Furthermore, bile salt molecules aggregate in helical, cylindrical, or spherical patterns which are strongly induced as a result of hydrogen bonding and ionic cross-linking [31]. In the present study, we expected more hydrogen bonding and ionic cross-linking due to the addition of Mnt and NaCl, which enhanced the gelation of the Na-DOC molecules. As shown in Figure 4, the crystallinity of the gel samples was assessed using counts (peaks) obtained from XRD analysis. The nature of the peaks in the XRD study is related to the crystallinity of the samples [44,49]. As shown in Figure 4c, the gel containing 50 mmole L^−1^ Mnt/NaCl displayed the highest number of peaks with the highest intensity. The six mean diffraction peaks observed at 27.47, 31.83, 45.54, 56.53, 66.29, and 75.36° were originated from NaCl nanocrystals, which correspond to the (111), (200), (220), (222), (400), and (420) planes, respectively, and the gel showed characteristic peak intensity (counts) of 2724 and 960 at 2θ ≈ 31.8 and 45.5°, respectively. This confirmed that the crystallinity of the hydrogel increased with Mnt/NaCl concentration.

The crystallinity and effect of NaCl on the gel structure of the sample prepared using 50 mmole L^−1^ Mnt and PBS at pH 6.5 was also tested. Unfortunately, as shown in Figure S4, the sample did not show any clear and distinct peak. This indicated that the sample existed as an amorphous gel [50] and that the halide salts (Na^+^ Cl^−^) played a significant role in cross-linking the microfibrils.

#### 3.1.3. Assessment of Hydrogen Bonding in the Gelation of Na-DOC Using FTIR

**Figure 5:**
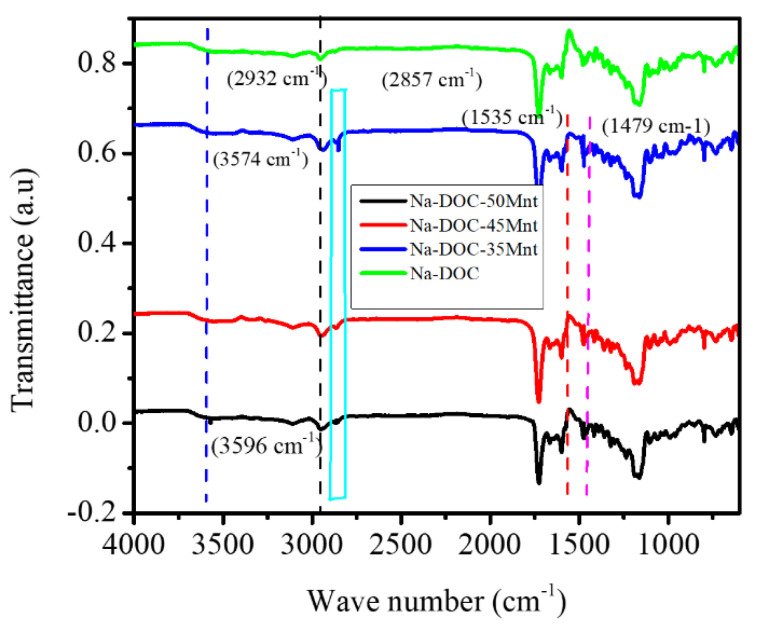
FTIR spectra of pure sodium deoxycholate (Na-DOC) and gels with 60 mmole L^−1^ Na-DOC and 35, 45 and 50 mmole L^−1^ concentration of Mnt/NaCl (1:1 mmole L^−1^ concentration ratio of Mnt/NaCl used).

FITR was used to confirm the effect of Mnt on the gelation of Na-DOC molecules. FTIR spectroscopy is used to assess hydrogen bond formation. In the FTIR spectra (Figure 5) the peak detected at 2857 cm^−1^ was assigned to the asymmetric stretching vibration of the C-H bond, and the asymmetric and symmetric methylene stretching bands were located at 2932 and 2857 cm^−1^, respectively. The stretching bands of the methylene units become less apparent when the concentration of Mnt decreased to 35 mmole L^−1^. Furthermore, the peaks at 1535 and 1475 cm^−1^ also correlated with the N-H vibration and the asymmetric vibration of CH_2_, respectively; these peaks weakened with reduction in Mnt/NaCl concentration [51]. This indicated that Mnt contributed maximally to hydrogen bond formation at its highest concentration.

#### 3.1.4. Na-DOC-Hyd Morphology Using FESEM

**Figure 6:**
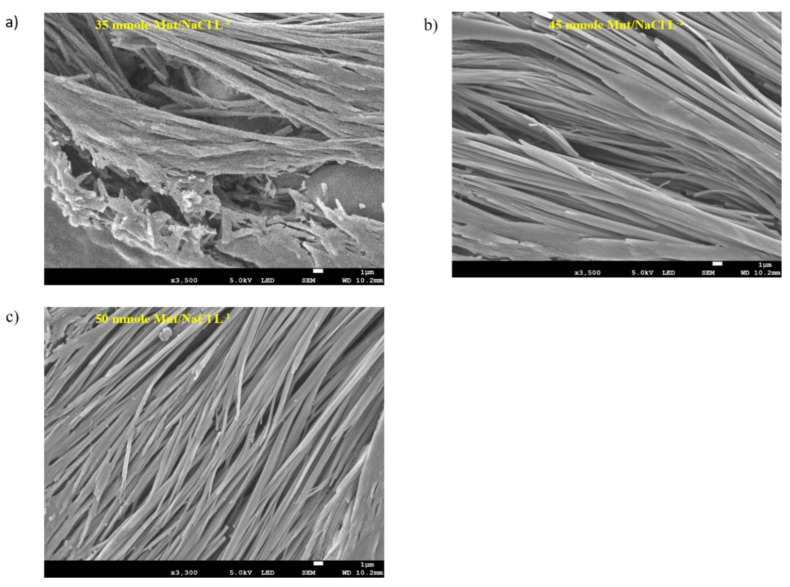
FESEM images of gels composed of 60 mmole L^−1^ Na-DOC and 35, 45, and 50 mmole L^−1^ Mnt/NaCl (1:1 mmole L^−1^ concentration ratio of Mnt/NaCl used).

The microstructure of the hydrogel samples with various Mnt/NaCl concentrations (35, 45 and 50 mmole L^−1^) were investigated using FESEM. The microscopic observation of the self-assembled microfibril structures may assist in understanding the macroscopic properties of the gels and allow interpretation of the mechanism of self-assembly or gelation of Na-DOC molecules [31,52,53]. As shown in Figure 6a–c, the gels prepared using 45 and 50 mmole L^−1^ Mnt/NaCl showed relatively better microfiber arrangement with noticeable perforations. Whereas as seen in Figure 6a, sample prepared using 35 mmole L^−1^ Mnt/NaCl showed deformed pores and the pores are have no regularity and the size widest of all samples. In particular, the gel formed using 50 mmole L^−1^ Mnt/NaCl showed network structure with intertwined microfibrils and relatively medium and homogenous interspersed pockets (Figure 6c). The pores observed between the intertwined microfibrils in the hydrogel enabled better drug loading and sustainable drug release. Studies have shown that this type of hydrogels allow controlled and persistent release of cancer drugs in local antitumor therapy. Based on the results of FESEM, we concluded that the incorporation of 50 mmole L^−1^ Mnt/NaCl possibly enhanced the assembly-driving force during gel formation in the Na-DOC molecules and consequently strengthened the hydrogel.

#### 3.1.5. Swelling Study of Na-DOC-Hyd

Drug loading and release using hydrogel depend on the hydrogel swelling capacity. In fact, most studies have shown that increase in the swelling ratio of the hydrogels increased the drug loading and release potential [54,55]. The results of the Na-DOC-hydrogel swelling studies showed that the swelling ratio depended on the NaCl concentration (Table S1). The calculated swelling ratio decreased with increase in NaCl concentration at pH 6.5. Hydrogel with 35 mmole L^−1^ Mnt/NaCl possessed swelling ratio of 22.94 ± 0.92. Increase in Mnt/NaCl concentration to 45 and 50 mmole L^−1^ in the same pH significantly decreased the swelling ratios to 15.96 ± 0.37 and 11.60 ± 0.23, respectively, after 7 h. In addition, as Mnt contributes significantly to hydrogen bond formation [44], the increase in Mnt concentration reduced the swelling capacity due to the presence of more physically cross-linked microfibrils in the sample.

#### 3.1.6. Degradation of Na-DOC-Hyd

Analysis of the degradation of the hydrogel at the highest Mnt/NaCl concentration revealed rapid degradation at the physiological pH of 7.4. This rapid degradation may be because the weak cross-linking density enhances buffer accessibility (erosion) and results in faster swelling and degradation [3]. The degradation of the Na-DOC hydrogel completed within 7 days in the tumor extracellular microenvironment (pH 6.5) (Figure S5). The information obtained from the degradation studies of the gels at pH 5.0 confirmed that the lower pH was not suitable for gel formation (Figure S6). Based on the result obtained from the degradation and swelling studies, we selected a sample generated using 50 mmole L^−1^ Mnt/NaCl for further drug loading and release study.

### 3.2. Drug Loading and Release Studies

#### 3.2.1. Doxorubicin Loading and Releasing from G4.5 PAMAM Dendrimer

Drug loading was performed by mixing the aqueous solution of G4.5 PAMAM dendrimer and DOX dissolved in DMSO at room temperature. Compared to the UV spectrum of free DOX, a red shift was observed in G4.5-DOX, indicating successful loading of DOX in G4.5 PAMAM dendrimer. The DEE and DLC were calculated to be 23.9% (*w/w*) and 7.21% (*w/w*), respectively.

The drug release of G4.5-DOX was assessed before entrapment into the Na-DOC-hydrogel along with the RESV, as the DOX was encapsulated within the G4.5 PAMAM dendrimer. DOX release occurred after degradation of both the Na-DOC-hydrogel and the G4.5 dendrimer polymer and after the release of some RESV. As the result, the release of DOX and RESV from the Na-DOC-hyd-RESV+G4.5-DOX will be in a controlled, sustainable, and sequential manner.

**Figure 7:**
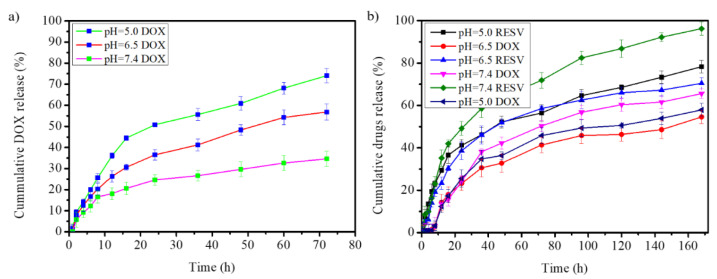
Cumulative drug release from (**a**) G4.5-DOX (DOX encapsulated in the G4.5 PAMAM dendrimer) and (**b**) Na-DOC-hyd-RESV+G4.5-DOX (G4.5-DOX entrapped in RESV-loaded Na-DOC-hydrogel) at pH 5.0, 6.5, and 7.4.

The release pattern of DOX from G4.5-DOX showed the highest cumulative DOX release of ∼74.07% at pH 5.0 (Figure 7a). Approximately 34.62% cumulative drug-release was observed at pH 7.4 after 72 h. This indicated that acidic pH increased drug release from the G4.5 dendrimer polymer. The highest DOX release was observed at pH 5.0, which might be due to the breakage of the weak electrostatic interactions between DOX and the polymer by the acidic pH, indicating that the G4.5 polymer is acid responsive. In addition, the cumulative drug release pattern showed a biphasic nature with an initial burst release, which accounted for > 46% of all the DOX released within the first 16 h at pH 5.0 (Figure 7a).

#### 3.2.2. Drugs Release from Na-DOC-Hyd-RESV+G4.5-DOX

As the Na-DOC molecules contribute to the amphiphilic nature of the gels [56], this may assist in the incorporation of the DOX-loaded G4.5 PAMAM, as well as RESV, in the space created between the intertwined microfibrils. Thus, preparation of dual drug co-loaded formulation in the Na-DOC-hydrogel is a reasonable approach. The G4.5-DOX was entrapped in RESV-loaded Na-DOC-hydrogel (Na-DOC-hyd-RESV) using simple mixing at the sol state. The concentration of DOX and RESV in the dual drug-loaded Na-DOC-hyd-RESV+G4.5-DOX were 0.4 mg mL^−1^ and 0.6 mg mL^−1^, respectively.

The, release of RESV and DOX from the Na-DOC-hyd-RESV+G4.5-DOX formulation was assessed under pH condition like used for G4.5-DOX (Figure 7a). The cumulative DOX and RESV release amounts were about 54.59% and 70.43%, respectively, after 7 days in an adjusted PBS at pH of 6.5 (Figure 7b). However, the cumulative percentages of DOX and RESV released at pH 7.4 were 70.43% and 96.23%, respectively, which were considerably higher. At the lower pH of 5.0, the rate of release of both DOX and RESV were 57.91% and 78.31%, respectively. The higher release of the drugs at pH 7.4 compared to that at pH 6.5 may be correlated with the higher deformation of the hydrogel at higher pH [37,57]. The higher RESV release from the hydrogel at pH 5.0 compared to that at pH 6.5 can be associated with pH responsiveness and precipitate formation of the Na-DOC molecule as shown in Figure 1 from gel formation and in Figure S5 from degradation study at lower pH 5.0.

As seen in Figure 7b, the cumulative drug release pattern indicated that the release rates of RESV at all pH were higher than that of DOX. This might be due to the loading of RESV on the Na-DOC-hydrogel, the high chance of RESV occurrence on the out surface of the hydrogel and due to the release of DOX requires degradation of both of the drug carriers (Na-DOC-hydrogel and the G4.5-PAMAM dendrimer). This release pattern indicated the presence of sequential and sustained drug release. This kind of drugs release pattern might be because of the presence of two carriers. The entrapped G4.5-DOX play a role to delay the release of DOX because the PAMAM polymer hold and delay the release of DOX until the hydrogel degraded to some extent and the polymer attacked by the acidic cancer microenvironment. As the result of the different localization of the two drugs in the two mixed carrier system the fast release of RESV followed by controlled release of DOX can be achieved. This sequential release may improve the therapeutic effect and cellular internalization of DOX. Literature also reported that enhancement of cellular uptake of DOX was achieved due to the inhibition of the activity of P-glycoprotein(P-gp) by the RESV. P-gp is an ATP-binding cassette protein found on the cell membrane that pumps many foreign substances including drugs out of cells [13,58].

To confirm the effect of the hydrogel on sustaining the sequence of drug release and lowering the release rate of DOX from Na-DOC-hyd-RESV+G4.5-DOX, the release pattern was compared with that of G4.5-DOX. As shown in Figure 7a,b, 56.82% DOX was released from G4.5-DOX in 72 h at pH 6.5, whereas 54.58% DOX was released from Na-DOC-hyd-RESV+G4.5-DOX in 7 days at the same pH. The rapid DOX release during G4.5-DOX study is due to the absence of the Na-DOC-hydrogel, which controls DOX release at a slow rate.

### 3.3. In Vitro Cytotoxicity Test (MTT Assay)

**Figure 8:**
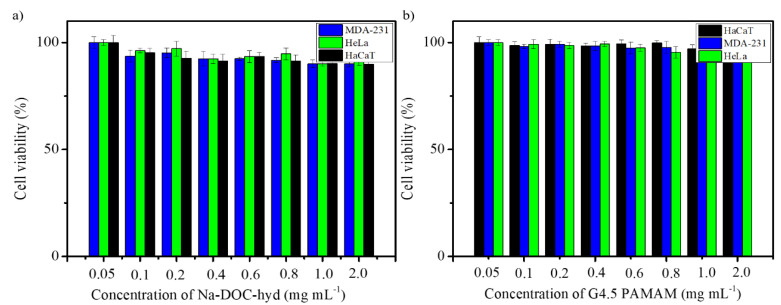
Cytotoxicity of various concentrations of (**a**) Na-DOC-hydrogel and (**b**) G4.5 poly(amidoamine) dendrimer in HaCaT, HeLa, and MDA-MB-231 cells.

Testing the biocompatibility of biomaterials in drug delivery systems is important prior to their application in animal models for assessing the efficacy of drug delivery. In agreement with this reasoning, the cytotoxicity of the empty G4.5 PAMAM dendrimer and Na-DOC-hydrogel were determined using the MTT cytotoxicity assay on three different cell lines. Cell viability was assessed based on optical absorbance at 570 nm using an ELISA plate reader. As shown in Figure 8a, the percentage of viable HeLa, MDA-MB-231, and HaCaT cells after incubation with maximum 2.0 mg mL^−1^ Na-DOC-hydrogel for 24 h were 91.23 ± 1.80, 90.06 ± 1.41%, and 89.96 ± 2.80%, respectively. The results of the biocompatibility study after co-incubation with maximum 2 mg mL^−1^ G4.5 PAMAM dendrimer revealed that the polymer was more biosafe than the Na-DOC hydrogel, as 98.23 ± 1.06, 96.68 ± 1.75, and 99.26 ± 2.06% viable cells were observed for HeLa, MDA-MB-231, and HaCaT, respectively (Figure 8b). These cytotoxicity studies confirmed that both the G4.5 PAMAM dendrimer and the hydrogel showed negligible toxicity towards different cells lines even at their highest doses. Thus, the biocompatibility studies of both the Na-DOC-hydrogel and G4.5 PAMAM dendrimer confirmed that the current drug delivery system conforms with the background information from the literature [27,28] and the results of former studies [37,38,40] and that it can be used safely in animal models as anticancer co-drug delivery system for DOX and RESV.

### 3.4. Cellular Uptake Studies

**Figure 9:**
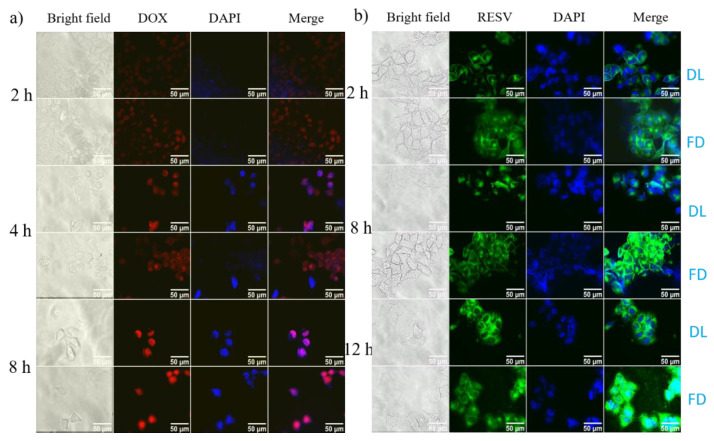
Fluorescence microscope images of HeLa cells incubated with (**a**) G4.5-DOX and (**b**) Na-DOC-hyd-RESV (in all treatment 2.5 µg mL^−1^ and 7.5 µg mL^−1^ equivalent concentration of DOX and RESV, respectively, were used), [DL = drug loaded materials treated cells (Na-DOC-hyd-RESV or G4.5-DOX) and FD = free drugs (RESV or DOX).

To evaluate the pH responsiveness of the biomaterials in G4.5-DOX NPS and Na-DOC-hyd-RESV formulations in cultured HeLa cells, the fluorescence intensity of the drugs released in the cells was monitored using fluorescent microscopy. After the HeLa cells were incubated with G4.5-DOX or Na-DOC-RESV for 2 h, the observed fluorescence of both DOX (red) and RESV (green) were weak (Figure 9a,b). However, the fluorescent intensity of the free drugs were higher (brighter) than those of the drug-loaded formulation (G4.5-DOX and Na-DOC-hyd-RESV) in HeLa cells at all time points. In addition, the fluorescence intensity of both drugs increased in a time-dependent manner. This difference in fluorescence intensity was also observed between free drug- and drug-loaded material-treated HeLa cells, which was strongly associated with the differences in the mechanism of cellular internalization of the free drugs and drug carriers [59]. As the time of treatment increased to 4 h and 8 h in G4.5-DOX-NP-treated cells, more DOX molecules were released, and bright red fluorescent intensity was observed in the cytoplasm and nuclei (Figure 9a). In contrast, greener fluorescence was observed in the cytoplasm and less in the nuclei for Na-DOC-hyd-RESV-treated HeLa cells even after 12 h of incubation (Figure 9b).

### 3.5. In Vivo Antitumor Study of Na-DOC-Hyd-RESV+G4.5-DOX

The in vivo synergistic antitumor effect of G4.5-DOX and RESV-loaded Na-DOC-hyd was assessed in HeLa cell xenograft tumors in BALB/c mice. The in-situ sol-to-gel phase change of the designed hydrogel at the pH of the tumor extracellular microenvironment was considered for sustained and sequential intratumoral drug release. The G4.5 PAMAM dendrimer and Na-DOC hydrogel underwent sequential, sustained, and controlled release of the two drugs at the tumor sites, the pH of which was mildly acidic (pH 6.5–6.7). During the study period, the change in tumor volume was measured to evaluate the tumor inhibition potential of the formulations.

**Figure 10:**
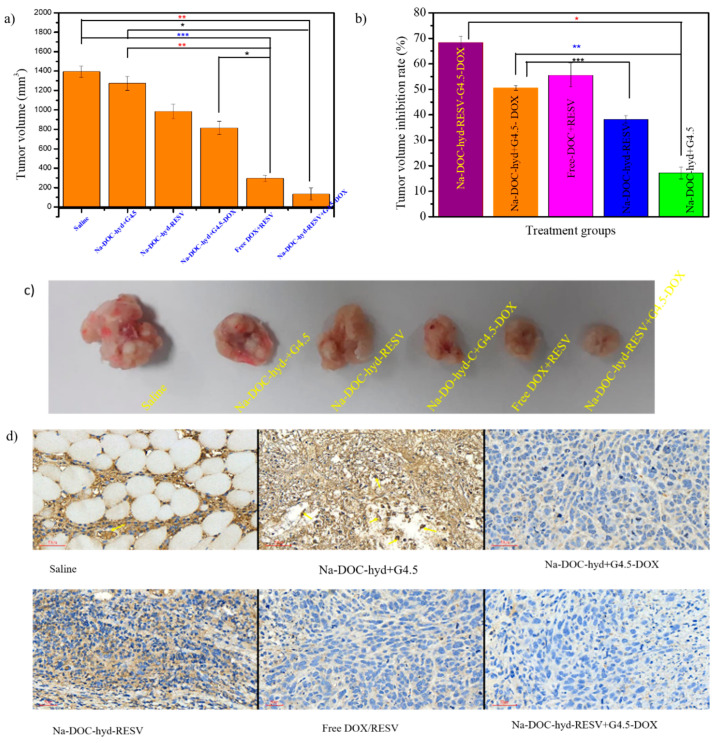
In vivo anti-tumor and ant angiogenic study in BALB/c nude mice inoculated with HeLa cells and treated with six different formulations. (**a**) Tumor volume, (**b**) the percentage of tumor volume inhibition rate (TVIR), (**c**) photograph of tumors samples collected from six different formulations treated mice groups, and (**d**) CD31-stained tumor tissue for estimating the micro-vessel density (MVD) (stained brown) after 28 days of treatment period (scale bar = 50 µm, * *p* < 0.05, ** *p* < 0.005, *** *p* < 0.001; *n* = 4).

As shown in Figure 10a, the tumor volume increased rapidly when tumor-bearing mice were treated with physiological saline (control) and Na-DOC-hyd+G4.5 (empty material). Na-DOC-hyd+G4.5-injected mice increased rapidly with time, indicating that saline and Na-DOC-hyd+G4.5 themselves do not possess any therapeutic potential for inhibiting tumor. Whereas, in those tumor-bearing mice treated with Na-DOC-hyd-RESV+G4.5-DOX and free RESV/DOX (at equivalent DOX and RESV concentration of 10 mg kg^−1^ and 30 mg kg^−1^, respectively) the tumor growth was significantly inhibited compared to that in saline-treated tumor-bearing mice. This might be linked to the synergistic effect of the two combined drugs because, RESV possess antitumor property and the ability to enhance cellular influx of DOX which in turn increased the anti-tumor properties of DOX [13].

Furthermore, the tumor volume of the Na-DOC-hyd-RESV and Na-DOC-hyd+G4.5-DOX-treated tumor bearing mice increased mildly, indicating that a single drug loaded hydrogel based formulation had lesser cancer cell killing potential than the dual drug formulations. However, all the drug-loaded formulation-treated groups were positively affected by either single or dual drugs, the highest tumor volume inhibition rate (TVIR) (68.37 ± 3.45) was observed in those tumor bearing mice group treated with Na-DOC-hyd-RESV+G4.5-DOX (Figure 10b).

In addition to testing the antitumor activity, the anti-angiogenic effects of the co-delivered DOX and RESV in the formulation were also investigated. Toward this, the samples collected from the tumor tissues (Figure 10c) of each group were stained using CD31 antibody for assessing micro-vessel density (MVD) and blood vessel size (diameter). As shown in Figure 10d, the tumor tissue collected from mice treated with Na-DOC-hyd-RESV+G4.5-DOX and free mixed DOX+RESV showed noticeable reduction in the density and diameter of the blood vessel (stained brown) compared to those mice treated with saline and Na-DOC-hyd+G4.5. This indicated that the presence of RESV along with DOX enhanced suppression of blood vessel formation and inhibited the growth of the already formed blood vessels, thereby reducing tumor volume. In contrast, the results obtained with single drug-loaded formulations, Na-DOC-hyd-RESV and Na-DOC-hyd+G4.5-DOX, were almost similar. Among the four drug based treatment groups, the reduction in MVD and size of the blood vessels in the Na-DOC-RESV+G4.5-DOX formulation-treated group were significant, which might be due to the synergistic [6] anti-angiogenic effect [13,15,60] of RESV.

In addition, compared to the free drugs, the Na-DOC-hyd-RESV+G4.5-DOX formulation strongly inhibited tumor growth, which might be due to the sequential, controlled, and sustainable release of the two drugs (DOX and RESV) at the tumor site. Owing to the relatively slow degradation of the Na-DOC-hyd drug carrier system, high concentrations of the drugs were maintained at the tumor site for more than a week. The tumor images obtained from the different formulation-treated groups also support the tumor growth pattern as well as the angiogenic nature of the tumor. As shown in Figure 10c, the smallest tumors with the least hemic appearance were collected from mice treated with Na-DOC-hyd-RESV+G4.5-DOX, whereas, the tumors obtained from the Na-DOC-hyd-RESV and Na-DOC-hyd+G4.5-DOX groups were more hemic and larger than those from mice treated with hydrogel loaded with both drugs. This indicated the synergistic effects of the combined DOX and RESV in anti-angiogenesis.

The H & E-stained images of tumor sections (Figure 10d) showed elongated cells with larger nuclei in the saline- and empty material-treated groups, indicative of the rapid proliferation of cancer cells. In contrast, tumor images of the Na-DOC-RESV+G4.5-DOX-treated group showed blurred staining and decreased tumor cell density with nuclear shrinkage, which confirmed the progression of tumor cell necrosis and enhanced tumor growth inhibition.

**Figure 11:**
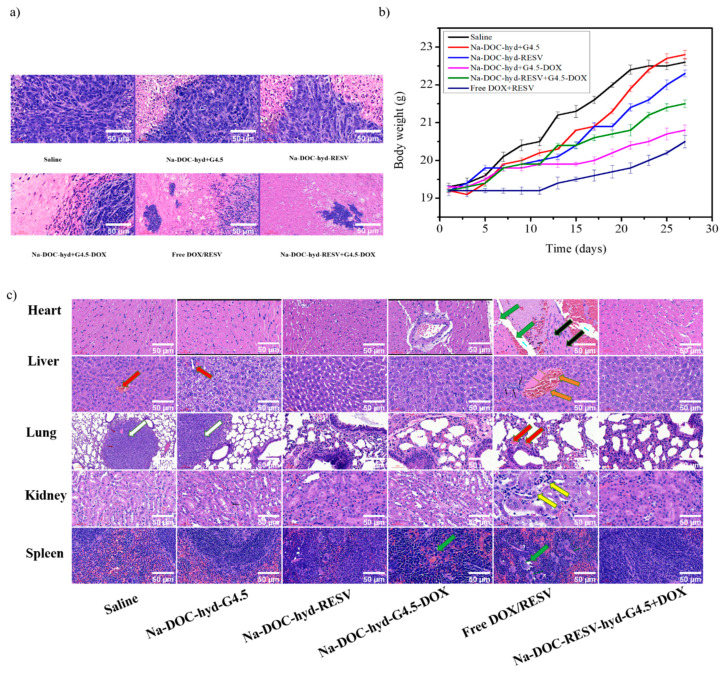
Assessment of biocompatibility in tumor-bearing mice using (**a**) histological analysis of necrotic and apoptotic areas in tumor tissue, (**b**) determination of body weight changes, and (**c**) histological analysis of major organs via hematoxylin (H) and eosin (E) staining after treatment of BALB/c mice with various formulations for 28 days.

The apoptotic and necrotic areas in the tumor samples collected from mice treated with the drug-loaded hydrogel based formulations (Na-DOC-hyd-RESV+G4.5-DOX, Na-DOC-hyd-RESV and Na-DOC-hyd+G4.5-DOX) were estimated to be 87.51 ± 5.42%, 71.45 ± 3.43, and 51.76 ± 2.67, respectively, which were considerably larger than those from mice treated with the empty hydrogel (23.99 ± 2.64%) and saline (< 5%) (Figure 11a). This proved that the hydrogel and the polymer do not have any biocompatibility-related issues and that the empty material does not possess any therapeutic property. Furthermore, the free RESV/DOX (54.97 ± 3.80%)-treated group showed larger necrotic area than the single drug-loaded hydrogel-based treated groups (Na-DOX-hyd-RESV and Na-DOC-hyd+G4.5-DOX), which could mainly be due to systemic toxicity of the freely diffused DOX in the area [61]. Based on the results of the in vivo model mice study, we concluded that the highest tumor growth inhibition was observed in mice treated with Na-DOX-RESV+G4.5-DOX, as this formulation offers synergistic antitumor effect due to sequential, controlled, and sustained release of the drugs at the tumor sites.

### 3.6. Histological Analysis of the Internal Organs

Although DOX possesses precise curative effects and is used to treat different forms of cancer as a first-line chemotherapeutic drug [2,62], it adversely affects normal cells by inducing cardiotoxicity and development of drug resistance, which has limited its application in cancer patients [2,63]. Several studies have been conducted to alleviate the undesired effects of DOX related to its toxicity. Combination of DOX with other chemicals, such as natural polyphenols, which is one of the approaches for improving its efficacy and reducing the limitations [64]. However, evaluating the biosafety of the drugs in combination therapy is crucial, especially in animal models. Based on this fact, the toxicity related to the different formulations in this study were evaluated by monitoring the changes in body weight and analyzing the histology of tumors and organs collected from tumor-bearing mice after 28 days of treatment. However, the survival rate of the mice during treatment was considered as an additional parameter, as all mice survived up to the end of the treatment period, because of which we could not incorporate any data on cytotoxicity-related death in any of the formulations.

As shown in Figure 11b, significant changes in body weight were observed in the free DOX/RESV and saline (control)-treated groups. The free DOX/RESV-treated group displayed the lowest body weight changes at the end of the treatment period. However, the differences in body weight of Na-DOC-hyd+G4.5 and Na-DOC-hyd-RESV-treated tumor-bearing groups were negligible compared to that of the control. This indicated that Na-DOC-hyd+G4.5 and RESV did not exert any pronounced acute or chronic toxic effects on mice body. The lowest body weight change in the free DOX/RESV-treated tumor-bearing group may be due to the toxicity of the high concentration and the free diffusion of DOX into the cells, which was cytotoxic.

To further investigate the systemic toxicity of various drug formulations, the histological changes in the major organs (liver, heart, kidney, lung, and spleen) of tumor-bearing mice were also analyzed. As shown in Figure 11c, mice treated with free DOX/RESV exhibited certain pathological manifestations on the organs such as mild focal myocytic degeneration, wide intercellular space (green arrow), and minimal mononuclear cell infiltration around the coronary artery (black arrow), and mononuclear inflammatory cell aggregation (black arrow) in the liver. Extramedullary hematopoiesis characterized by prominent increase in the number of megakaryocytes and erythroblasts and decrease in lymphocyte number due to loss of lymphoid follicle structures (green arrow) in the spleen were observed. However, the parietal epithelial cells of the Bowman’s capsule of the kidney were replaced by cuboid cells, a feature termed metaplasia (yellow arrow), along with aggregation of tumor cells (white arrow) in the lungs in mice treated with saline. Mild increase in hepatocytic mitosis (orange arrow) was observed in the liver of mice treated with free drugs. The abnormal histological symptoms detected (white arrow) in the lung and liver of mice treated with saline and empty Na-DOC-hyd+G4.5 biomaterial indicated the possibility of metastasis in mice not treated with any therapeutic drugs [65,66]. In contrast, organs collected specifically from mice treated with the Na-DOC-hyd-RESV+G4.5-DOX formulation did not show any significant histological abnormality, confirming the biosafety of the drug carriers, as well as RESV. Furthermore, the slow and sustained release of DOX (because of the G4.5 dendrimer polymer and Na-DOC-hyd) and the role of RESV in modulating the cytotoxicity of DOX, contributed considerably to the better biocompatibility attributes of the formulation. The pH responsive materials has been designed to be stable in the extracellular cancer environment, which allow to maintain the loaded drugs in the cancer environment for longer time and slowly releases the RESV and DOX in a sequential and sustainable manner. Because those mice treated with dual drug-loaded hydrogel showed the highest antitumor and anti-angiogenesis effect, which indicated that this controlled and persistent drug release effects has been observed on the formulation of Na-DOC-hyd-RESV+ G4.5-DOX. However, the thixotropy nature of the hydrogel need to be improve for maintaining and release of the drug for much longer than 7 days in the local drug delivery of the intratumor areas.

## 4. Conclusions

In this study, an injectable in situ Na-DOC-hydrogel was prepared at the extracellular pH of cancer cells for controlled and sequential release of DOX and RESV from co-encapsulated of RESV and G4.5-DOX NPs. This Na-DOC-hyd-RESV+G4.5-DOX formulation maintained suitable DOX and RESV concentration at the local tumor site for relatively longer periods compared to the G4.5-DOX alone, and the formulation reduced systemic DOX exposure to normal tissues. Furthermore, the synergism between DOX and RESV therapies resulted in high therapeutic efficacy against HeLa cell tumor xenografted in BALB/c nude mice. A synergistic anticancer effect of DOX and RESV combination therapy was observed in vivo in tumor-bearing mice. The Na-DOC-hyd showed higher loading capacity and released the drugs (DOX/RESV) in a sequential, sustained, controlled, and pH-dependent manner. Both the G4.5-PAMAM dendrimer and Na-DOC-hyd drug carrier displayed good cytocompatibility with MDA-MB-231, HeLa, and HaCaT cells and exhibited negligible systemic toxicity in vivo in a mouse model. However, the thixotropy nature of the hydrogel need to be improve more for maintaining and release of the drug for much longer than 7 days of degradation in the local drug delivery.

## Supplementary Materials

The following are available online at https://www.mdpi.com/1999-4923/12/11/1069/s1, Figure S1: Spectroscopic absorbance vs wavelength and (a) sample concentration for Calibration curve preparation for mixed drugs (DOX/RESV) (b) DOX loaded G4.5PAMAM dendrimer (c) RESV loaded Na-DOC-hyd (d) Standard curve/Calibration curve, Figure S2: Test tube inversion study of phase change and stability behavior of gels samples made of 60 mmole L^−1^ sodium deoxycholate (Na-DOC) with 30, 35, 40 and 50 mmole L^−1^ concentration of Mnt/NaCl), Figure S3: Complex modulus (G*) as a function of the applied stress at 1.0 Hz; for samples of gels with of 60 mmole L^−1^ sodium deoxycholate(Na-DOC) and 35, 45 and 50 mmole L^−1^ concentration of Mnt/NaCl (1:1 mmole L^−1^ concentration ratio of Mnt/NaCl used). The lines are guides for the eyes, Figure S4: XRD patterns of the gels with 60 mmole L^−1^ sodium deoxycholate (Na-DOC) and 50 mmole L^−1^ concentration of mannitol (Mnt), Figure S5: Degradation behavior of gel made of 60 mmole L^−1^ sodium deoxycholate (NaDOC) with 35, 45 and 50 mmole L^−1^ concentration of Mnt/NaCl) in PBS at pH of 6.5, Figure S6: Gel nature during degradation and swelling test a) gel form precipitate in PBS at pH of 5.0 b) gel residual after degradation in PBS at pH 6.5, Table S1: Swelling ratio of gel made of 60 mmole L^−1^ sodium deoxycholate (Na-DOC) with 30, 45 and 50 mmole L^−1^ concentration of Mnt/NaCl) in PBS at pH of 6.5.
