# Supplementary material for: Bioinspired Composite, pH-Responsive Sodium Deoxycholate Hydrogel and Generation 4.5 Poly(amidoamine) Dendrimer Improves Cancer Treatment Efficacy via Doxorubicin and Resveratrol Co-Delivery
Source: Pharmaceutics. 2020 Nov 9;12(11):1069. doi: 10.3390/pharmaceutics12111069 (PMC7696475; doi:10.3390/pharmaceutics12111069)
Supplement: Supplementary file 1 [file pharmaceutics-12-01069-s001.pdf]

pharmaceutics

Supplementary Materials: Bioinspired Composite, pH-Responsive Sodium Deoxycholate Hydrogel and Generation 4.5 Poly(amidoamine) Dendrimer Improves Cancer Treatment Efficacy via Doxorubicin and Resveratrol Co-Delivery

Tefera Worku Mekonnen, Abegaz Tizazu Andrgie, Haile Fentahun Darge, Yihenew Simegniew Birhan, Endiries Yibru Hanurry, Hsiao-Ying Chou, Juin-Yih Lai, Yen-Hsiang Chang, Hsieh-Chih Tsai * and Jen Ming Yang *

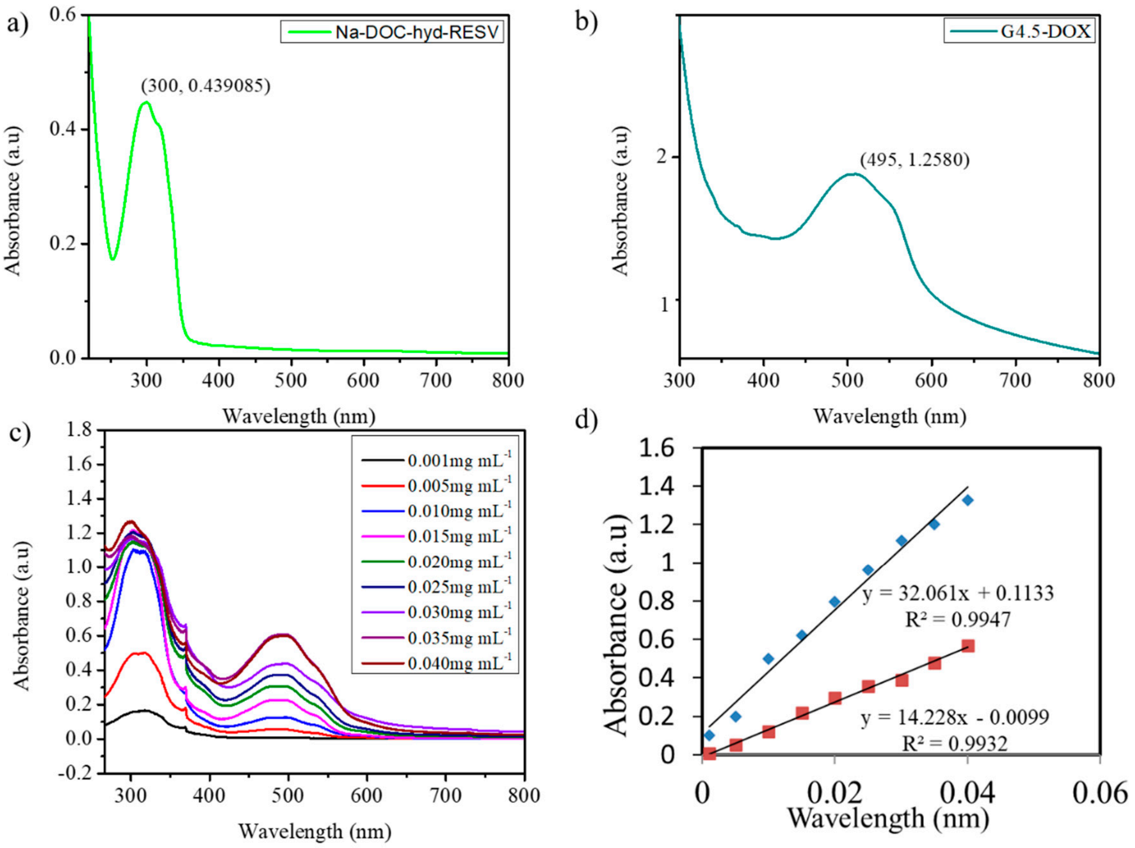

Figure S1. Spectroscopic absorbance vs wavelength and (**a**) sample concentration for Calibration curve preparation for mixed drugs (DOX/RESV) (**b**) DOX loaded G4.5PAMAM dendrimer (**c**) RESV loaded Na-DOC-hyd (**d**) Standard curve/Calibration curve.

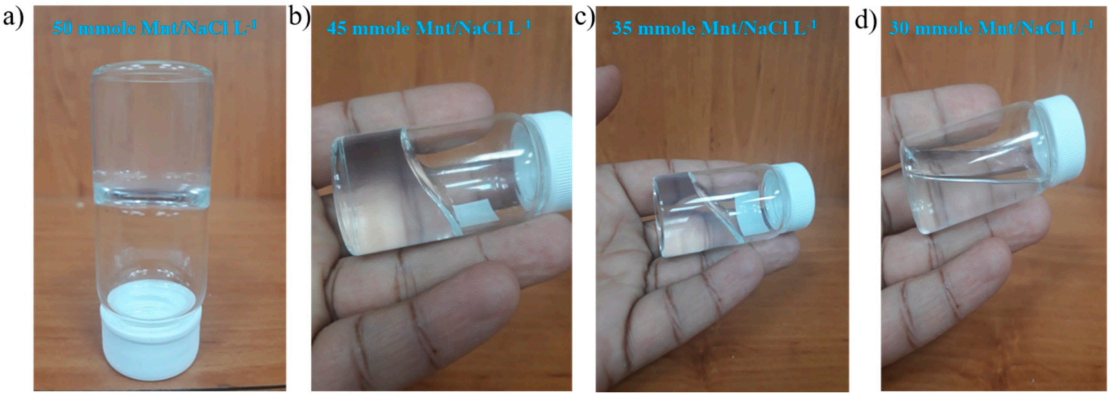

Figure S2. Test tube inversion study of phase change and stability behavior of gels samples made of 60 mmole L^{-1} sodium deoxycholate (Na-DOC) with 30, 35, 40 and 50 mmole L^{-1} concentration of Mnt/NaCl).

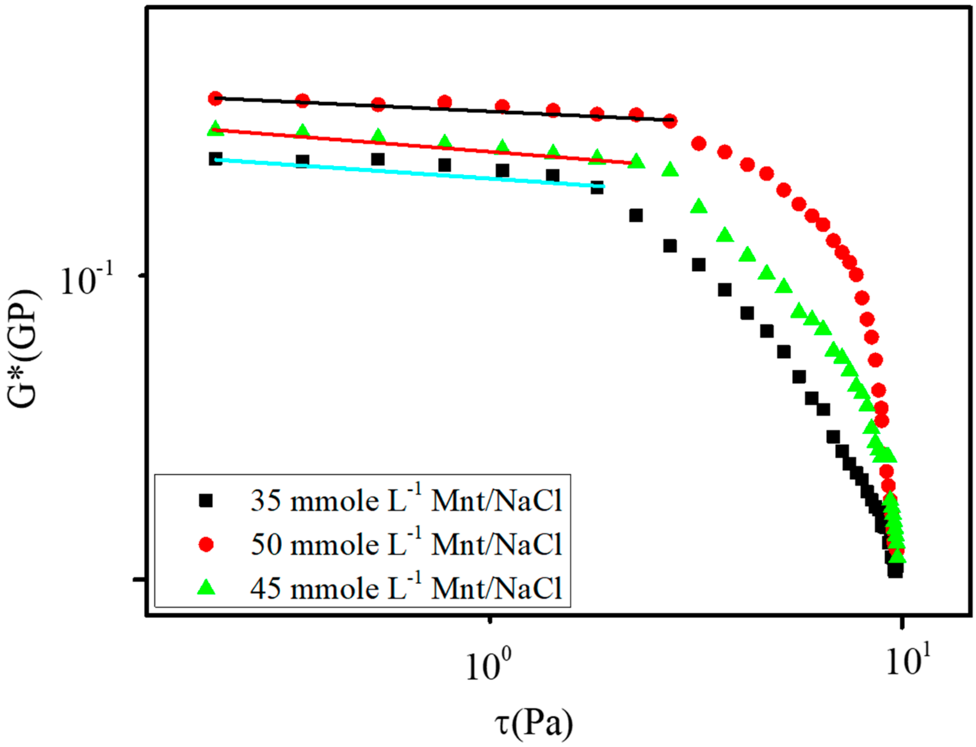

Figure S3. Complex modulus (G*) as a function of the applied stress at 1.0 Hz; for samples of gels with of 60 mmole L^{-1} sodium deoxycholate(Na-DOC) and 35, 45 and 50 mmole L^{-1} concentration of Mnt/NaCl (1:1 mmole L−1 concentration ratio of Mnt/NaCl used). The lines are guides for the eyes.

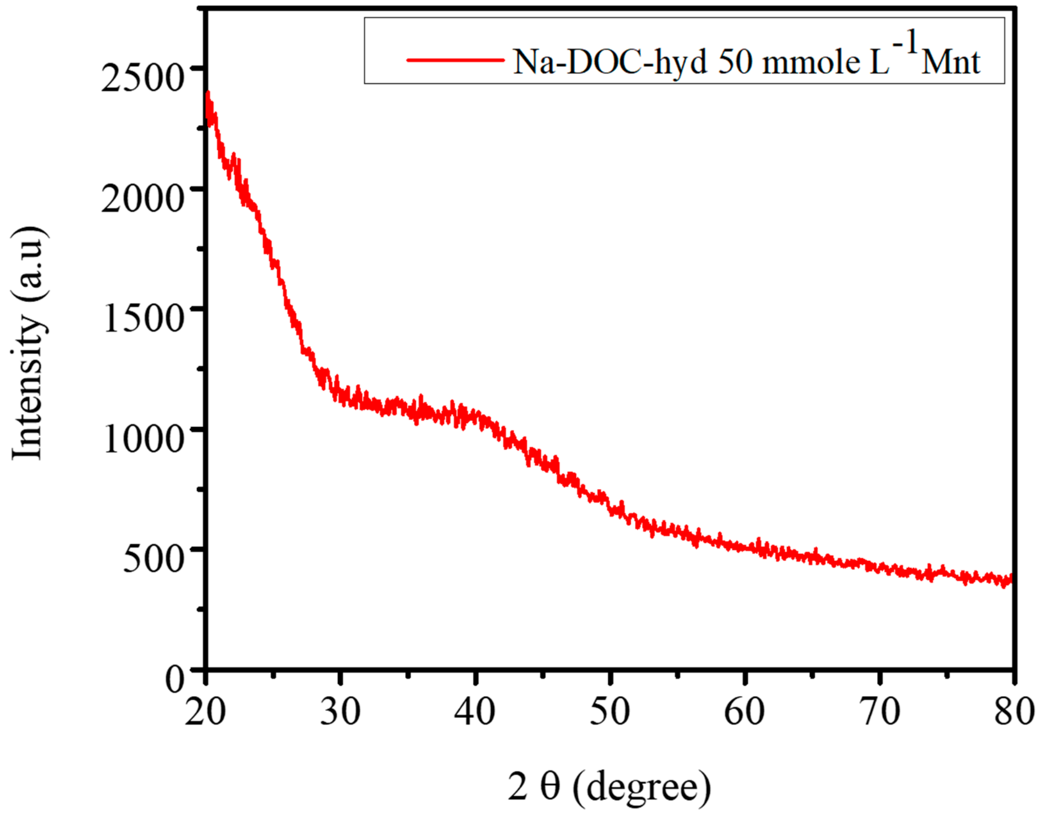

Figure S4. XRD patterns of the gels with 60 mmole L⁻¹ sodium deoxycholate (Na-DOC) and 50 mmole L⁻¹ concentration of mannitol (Mnt).

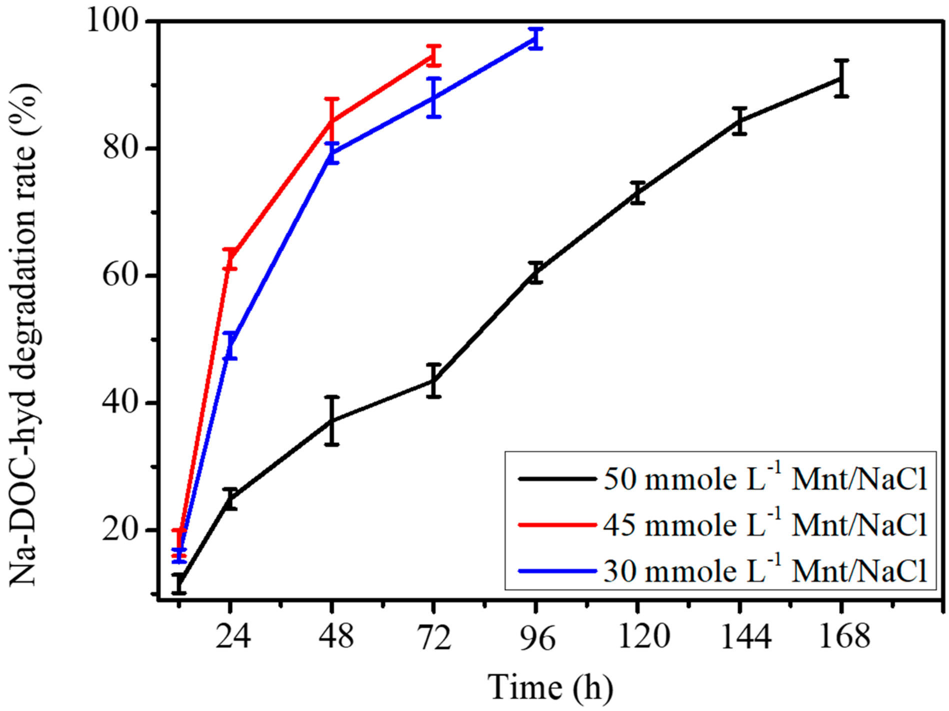

Figure S5. Degradation behavior of gel made of 60 mmole L⁻¹ sodium deoxycholate (NaDOC) with 35, 45 and 50 mmole L⁻¹ concentration of Mnt/NaCl) in PBS at pH of 6.5.

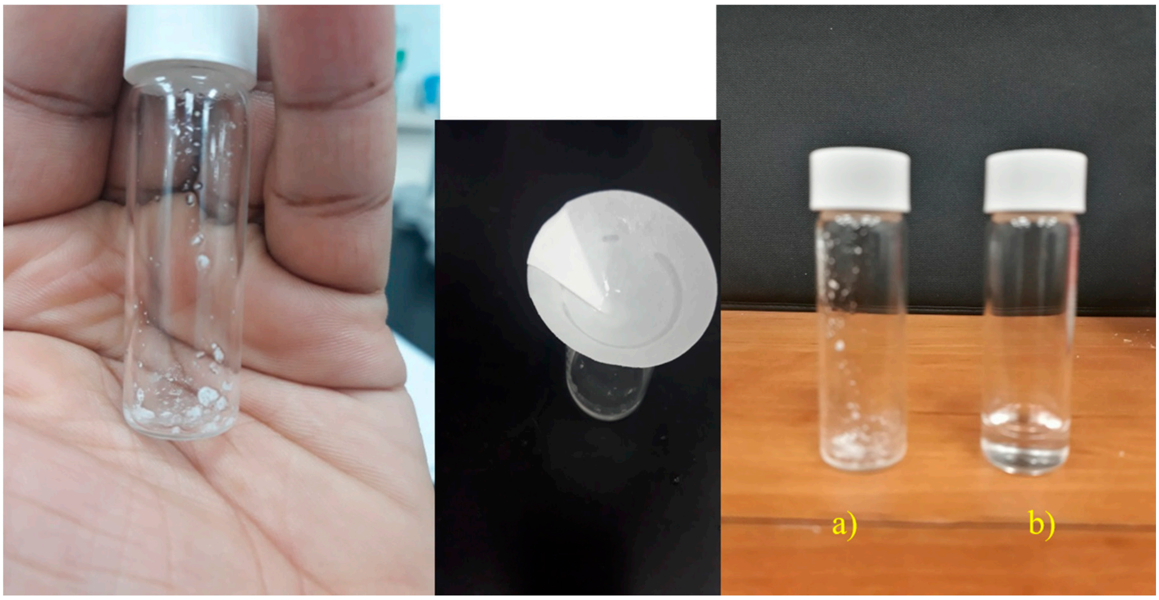

Figure 6. Gel nature during degradation and swelling test **a**) gel form precipitate in PBS at pH of 5.0 **b**) gel residual after degradation in PBS at pH 6.5.

Table 1. Swelling ratio of gel made of 60 mmole L^{-1} sodium deoxycholate (Na-DOC) with 30, 45 and 50 mmole L^{-1} concentration of Mnt/NaCl) in PBS at pH of 6.5.

Mnt/NaCl (mmole L^{-1})	Swelling ratio
35	22.94 ± 0.92
45	15.96 ± 0.37
50	11.60 ± 0.23
